# Current Prophylaxis and Treatment Approaches for Acute Graft-Versus-Host Disease in Haematopoietic Stem Cell Transplantation for Children With Acute Lymphoblastic Leukaemia

**DOI:** 10.3389/fped.2021.784377

**Published:** 2022-01-06

**Authors:** Matthias Wölfl, Muna Qayed, Maria Isabel Benitez Carabante, Tomas Sykora, Halvard Bonig, Anita Lawitschka, Cristina Diaz-de-Heredia

**Affiliations:** ^1^Pediatric Hematology, Oncology and Stem Cell Transplantation, Children's Hospital, Würzburg University Hospital, Würzburg, Germany; ^2^Aflac Cancer and Blood Disorders Center, Children's Healthcare of Atlanta, Emory University, Atlanta, GA, United States; ^3^Department of Pediatric Hematology and Oncology, Hospital Universitari Vall d'Hebron, Vall d'Hebron Institut de Recerca (VHIR), Barcelona, Spain; ^4^Haematopoietic Stem Cell Transplantation Unit, Department of Pediatric Haematology and Oncology, Comenius University Children's Hospital, Bratislava, Slovakia; ^5^Institute for Transfusion Medicine and Immunohematology, Goethe-University Frankfurt/Main, Frankfurt, Germany; ^6^German Red Cross Blood Service BaWüHe, Frankfurt, Germany; ^7^Department of Pediatrics, St. Anna Kinderspital and Children's Cancer Research Institute, Medical University of Vienna, Vienna, Austria

**Keywords:** acute graft-versus-host disease (aGVHD), management, hematopoietic (stem) cell transplantation, children, acute lymphoblastic leukaemia

## Abstract

Acute graft-versus-host disease (aGvHD) continues to be a leading cause of morbidity and mortality following allogeneic haematopoietic stem cell transplantation (HSCT). However, higher event-free survival (EFS) was observed in patients with acute lymphoblastic leukaemia (ALL) and grade II aGvHD vs. patients with no or grade I GvHD in the randomised, controlled, open-label, international, multicentre Phase III For Omitting Radiation Under Majority age (FORUM) trial. This finding suggests that moderate-severity aGvHD is associated with a graft-versus-leukaemia effect which protects against leukaemia recurrence. In order to optimise the benefits of HSCT for leukaemia patients, reduction of non-relapse mortality—which is predominantly caused by severe GvHD—is of utmost importance. Herein, we review contemporary prophylaxis and treatment options for aGvHD in children with ALL and the key challenges of aGvHD management, focusing on maintaining the graft-versus-leukaemia effect without increasing the severity of GvHD.

## Introduction

Relapse is the primary cause of failure of haematopoietic stem cell transplantation (HSCT) for paediatric acute lymphoblastic leukaemia (ALL). Results obtained in the randomised, controlled, open-label, international, multicentre Phase III For Omitting Radiation Under Majority age (FORUM) trial showed a higher probability of leukaemia-free survival in patients aged 4–21 years at HSCT with ALL experiencing grade II acute graft versus host disease (aGvHD) vs. patients with no or grade I GvHD, thus suggesting that moderate-grade aGvHD is associated with a graft-versus-leukaemia (GvL) effect protecting against leukaemia recurrence (1). However, unchecked aGvHD continues to be the leading cause of morbidity and mortality following HSCT (2). For patients with ALL and a high risk of relapse, HSCT is a key element to establish long-term control based on GvL activity (3). However, severe aGvHD (grade III–IV) needs to be avoided because it has the potential for life-threatening consequences (4), being even more deleterious in children than in adults as the sequelae occur within a delicate interplay of many developing organ systems in the growing child (5). Furthermore, it has to be kept in mind that aGvHD is the main risk factor for developing chronic graft versus host disease (cGvHD) (6, 7).

Herein, we review aspects of aGvHD pathology and management especially relevant to paediatric patients and the treatment of high-risk leukaemia. We explore approaches to GvHD prophylaxis, diagnosis and grading, and the incorporation of GvHD biomarkers into risk stratification models and response assessment. In addition, we discuss the key challenges and evidence surrounding aGvHD and the GvL effect.

## Clinical Evidence for a Graft-Versus-Leukaemia Effect as the Decisive Factor For Allo-Transplantation in All

The FORUM study was just the latest to indicate that higher survival may be linked to the presence of a moderate degree of aGvHD, and a number of studies provide clinical evidence for the presence of a GvL effect in ALL (8–16). These studies suggested an effect of both acute and chronic GvHD in decreasing leukaemia relapse.

An early Italian study in children with ALL showed how GvHD prophylaxis impacted the relapse rate in children with ALL in second remission given HSCT from unrelated donors (11). The rate was 0% for patients who received cyclosporine + methotrexate vs. 72% for those who received cyclosporine + methotrexate + Campath (*p* = 0.0002). Patients with grade II aGvHD presented higher EFS rate 64% (95% CI 40–88) than those with grade 0-I 36% (95% CI 14–58) and grade III-IV 29% (95% CI 8–51) (*p* = 0.04). Another Italian study determined a probability of relapse for children with ALL and cGVHD of 14% (95% CI, 6–21%) compared to 47% (95% CI, 39–54%) for children with ALL but without cGVHD (*p* = 0.0001) (12). Gustafsson Jernberg et al. showed in a single centre study that cGvHD had a significant impact on relapse (30% in patients with cGvHD vs. 53% in patients with no cGvHD) and survival (76% in patients with cGvHD vs. 45% for those with no cGvHD) (13). Later, the AIEOP-HSCT group demonstrated that grade III aGvHD vs. no aGvHD protected against leukaemia relapse (RR 0.32, *p* = 0.019) and improved EFS (RR 0.46, *p* = 0.047). Limited cGvHD vs. no cGvHD also impacted relapse rate (RR 0.42, *p* = 0.026) (14).

In the phase 3 Children's Oncology Group/Paediatric Blood and Marrow Transplant Consortium trial (ASCT0431) Pulsipher et al. showed that a grade I-III aGvHD had an independent effect decreasing leukaemia relapse. Grades I-III aGvHD led to a HR of 0.44 for relapse compared with no aGvHD (*P* = 0.04) and two fold improvement in EFS (*p* = 0.01). *De novo* cGvHD did not impact outcomes, but cGvHD ocurring after aGvHD protected against leukaemia relapse (HR 0.14) compared with no GvHD (*p* = 0.05). Moreover, it was shown that patients who were MRD+ pre-transplant and developed aGvHD in the first 2 months after HSCT did not relapse (15). Consequently, patients who do not develop aGvHD in the first 2 months are candidates for rapid withdrawal of immunosupression and potential candidates for other interventions such as post-transplant immune modulation and other immunotherapeutic approaches (17–20). Bader et al. had previously shown that rapid withdrawal of immunosupression can be safely performed in high-risk population with important improvement in survival (21).

More recently in a landmark analysis that combined MRD measurements after HSCT and aGvHD both were clearly associated with EFS and relapse (*p* < 0.001) (16). Patients who did not present aGvHD had a higher relapse incidence and lower EFS than those who developed aGvHD in both MRD positive or negative groups. For patients with detectable post-transplant MRD at day +30, but also at day + 90 and +180 the development of aGvHD led to a significant decrease in relapse rate and an improvement in EFS, providing evidence that GvHD/GvL can be beneficial to these patients. In addition, relapse was a rare event in patients who were MRD negative and developed aGvHD. On the other hand, this study highlighted that grade IV GvHD is not beneficial, and in consequence physicians should be cautious about interventions that stimulate excessive GvHD.

## Risk Factors for the Development of AGvHD in Children and Adolescents

The management of GvHD in patients with haematologic malignancies undergoing HSCT carries the additional challenge of maintaining the GvL effect while keeping GvHD at bay. Identifying patients at low and high risk of GvHD helps to establish GvHD prophylaxis: attenuating the intensity of GvHD prophylaxis for patients at lower risk of GvHD could mitigate the risk of relapse. On the contrary, patients at high risk of GvHD need more intense prophylaxis.

Well-known risk factors for the development of aGvHD—such as the use of mismatched and unrelated donors (22–27), a female-to-male donor-to-recipient constellation (28), the use of total body irradiation (TBI)-containing conditioning regimens rather than chemotherapy-based regimens (29), and the higher donor-age (30, 31)-have a major impact on the development of aGvHD. Regarding the stem cell source there are no randomised studies that compared peripheral blood stem cell (PBSC) vs. bone marrow transplants. A metanalysis showed that aGvHD was slightly increased (RR 1.16, *p* = 0.006) and chronic GvHD was increased (RR 1.53, *p* ≤ 0.001) when comparing PBSC and bone marrow transplants (32), however there are few reports of acute GvHD following PBSC transplants in paediatrics. In children and young adults, age-related factors are likely to affect outcomes even more than in adults (33): for example, the pharmacokinetics of many drugs (including chemotherapy for conditioning or immunosuppression) vary among very young children even when properly adjusted for body surface or weight (25, 34). Thymic function is another prime example: it gradually decreases in adolescents leading to delayed immune reconstitution (35) (see paper by Eyrich et al. in another review in this research topic section). Unfortunately, data on such underlying risk factors are consistently under-reported or have been unavailable for analysis, making comparisons between clinical studies difficult.

## Differences in the Pathophysiology of AGvHD Between Adults and Children

Acute GvHD is the sum of the allo-reactive immunologic activity of the graft directed against the healthy tissue of the host. The pathophysiology of GvHD is as complex as the regulation of the immune system itself, but the principles for the initiation of aGvHD, manifesting classically in the skin, gut and liver, often are summarised as a cycle of self-perpetuating events (2): T cells derived from the graft interact with residual patient antigen-presenting cells and epithelial allo-antigens resulting in a self-perpetuating cycle of activation, release of inflammatory mediators and further activation (9). Tissue damage is generally considered to be higher when total body irradiation is part of the conditioning regimen (as is standard for children with ALL ≥4 years) than with chemoconditioning alone (36, 37). Loss of the mucosal barrier, translocation of gastrointestinal bacteria and lack of regulatory mechanisms due to conditioning lead to the activation of transplanted donor T cells, which subsequently proliferate rapidly and traffic into the periphery, ultimately causing target organ damage. While these principal mechanisms may be the key sequence for most cases of GvHD, independent of age, it is conceivable that pathophysiology in children greatly differs from adults.

In a large retrospective study including over 5,000 adult patients, Jagasia et al. found that, among 2,370 adult patients transplanted from an unrelated donor, one third developed aGvHD of grade C and D (approximately corresponding to grade III and IV) (38). A third of the patients had transplant-related mortality (TRM) within 1 year, suggesting that higher grade GvHD in adults has a dismal prognosis. In contrast, in the tightly controlled cohort of paediatric ALL patients included in the FORUM study, 43 (10.8%) out of 396 evaluable patients developed severe aGvHD (1). Two-year TRM of all evaluable patients was 5.8%. A retrospective study of 476 paediatric patients with leukaemia also demonstrated an age-dependent risk of severe GvHD: compared with age 13–18 years, age 2–12 years was associated with a lower risk of grade II–IV aGvHD [hazard ratio (HR), 0.42; 95% confidence interval (CI), 0.26 to 0.70; *p* = 0.0008], grade III–IV aGvHD (HR, 0.24; 95% CI, 0.10–0.56; *p* = 0.001), and cGvHD (HR, 0.32; 95% CI, 0.19–0.54; *p* < 0.001) (33). These findings suggest that children undergoing HSCT might be at lower risk of severe GvHD—and subsequently of TRM—than are adults, although the reasons for this are not fully understood.

Two key differences in children vs. adults are undisputed: overall co-morbidity in children is lower resulting in better organ function and tolerance of potentially toxic drugs and secondly thymic function is generally better (35), as it linked to age and the hormonal status (39). In consequence, T-cell recovery of T-cells “educated” in the thymus is faster. Wound healing and organ recovery is improved. Furthermore, differences in the pharmacokinetics of immunosuppressive drugs (40) and differences in gut microbiome (41) have been described. Data on differences in the paediatric vs. the adult population are emerging from studies investigating how a paediatric-like treatment protocol works in younger adult patients with leukaemia and lymphoma: multiple factors related to biology but also therapy intensity affects the balance between tolerability and efficacy (42–44). These differences in pathophysiology by patient age have to be kept in mind when children are transplanted in clinical units, where both, adults and children are being treated, or when results of clinical trials performed predominantly in adult populations are used as the basis for clinical decisions in children.

In addition to the three typical organs involved in aGvHD (the skin, liver, gastrointestinal tract), other tissues such as the thymus, bone marrow and secondary lymphoid organs may be involved. This is of importance in the context of impaired haematopoiesis, immune reconstitution, and subsequent cGvHD (45).

## Prophylaxis of GvHD in Different Settings of HSCT

Currently, GvHD prophylaxis is often based on a calcineurin inhibitor such as cyclosporine A or tacrolimus with or without a short course of methotrexate (46). Both tacrolimus and cyclosporine A reduce T-cell function via inhibition of calcium-dependent signal transduction downstream of the T-cell receptor (TCR). Two large multicentre studies conducted mainly in adults have shown the superiority of tacrolimus over cyclosporine A in reducing aGvHD, with no difference in overall survival (OS) and relapse-free survival (47, 48).

Calcineurin inhibitors are associated with various toxicities such as renal dysfunction, neurological side effects and transplant-associated thrombotic microangiopathy. The dosing is typically adjusted to maintain a therapeutic level while avoiding toxicities but the target concentration is still a matter of debate. The updated European Society of Bone and Marrow Transplantation (EBMT) consensus guidelines recommend a cyclosporine A target concentration of 200–300 ng/mL in the first 4 weeks, followed by 100–200 ng/mL for adult patients undergoing standard-risk human leukocyte antigen (HLA) matched HSCT (46). A retrospective study in paediatric patients reported a strong relationship between cyclosporine A blood levels during the first 2 weeks post transplantation and the severity of aGvHD. A cyclosporine A level of >120 ng/mL was more protective than levels below this threshold (31, 49). The EBMT Paediatric Diseases Working Party (PDWP) survey found that after myeloablative conditioning for a matched sibling donor (MSD) HSCT, single-agent cyclosporine A was used in half of participating centres, and 85% of centres aimed for a blood concentration of 100–200 ng/mL within the first 8 weeks post transplantation. The median duration of cyclosporine A prophylaxis was 110 days [interquartile range (IQR) 90 days], with the majority of centres adjusting both duration and blood level based on each patients' estimated risk of relapse. The use of bone marrow vs. peripheral blood mononuclear cells as the stem cell source did not influence the approach to calcineurin-inhibitor–based prophylaxis in 73% of responding centres (50).

Co-administration of methotrexate with a calcineurin inhibitor reduces the risk for cGvHD and aGvHD; however, when bone marrow is used as the stem cell source, monotherapy with cyclosporine A may be considered for MSD HSCTs (51). Methotrexate is a folic acid antagonist and antimetabolite that mitigates T-cell activation at low doses (52). A recent EBMT PDWP survey found that many participating centres reported using monotherapy with cyclosporine A for bone marrow as the graft source; those centres using methotrexate typically applied three doses of 10 mg/m^2^ on days +1, +3, and +6 followed by folic acid rescue (50).

Because of the favourable toxicity profile of mycophenolate mofetil (a selective inhibitor of inosine monophosphate dehydrogenase), the EBMT recommends its use instead of methotrexate in patients with contraindications to methotrexate or in patients receiving reduced-intensity conditioning prior to HSCT and in cord blood transplants. However, comparative evidence in children for mycophenolate mofetil vs. methotrexate is lacking.

In contrast to calcineurin inhibitors, sirolimus—an oral mammalian target of rapamycin (mTOR) inhibitor—suppresses the expansion of conventional T cells more potently than the expansion of regulatory T cells (53). Sirolimus has demonstrated activity for the prevention of aGvHD in adults with and without the combination of tacrolimus and methotrexate (14). A randomised study conducted in 209 patients including 24 patients <18 years old with malignant and non-malignant diseases undergoing HSCT compared GvHD prophylaxis with cyclosporine A plus methotrexate vs. sirolimus plus tacrolimus, and concluded that the combination sirolimus plus tacrolimus was valid and safe, however the number of paediatric patients was small (54).

Anti-thymocyte globulin (ATG) is the purified polyclonal immunoglobulin G (IgG) fraction from the sera of horses or rabbits immunised with human thymocytes or T-cell lines. It is applied for *in vivo* pan-T-cell depletion. ATG has been demonstrated to reduce the incidence of aGvHD and cGvHD when added to standard prophylaxis prior to HSCT (55, 56). The EBMT recommends the use of ATG in matched unrelated donor (MUD) HSCT and in MSD HSCT where the risk for the development of GvHD is high (46). There is evidence that the pharmacokinetics and subsequent ATG serum levels post-HSCT affect the degree of immunosuppression possibly affecting non-relapse mortality (NRM) and relapse. Individualised ATG dosing based on absolute lymphocyte count, as a corrective of the weight-adjusted dosing, could be a way to control the risk of GvHD without impairing NRM and relapse (57). A detailed review regarding the use of serotherapy is provided by Koegh and colleagues in another review in this topic research section.

Alemtuzumab is a humanised monoclonal antibody against CD52 which is predominantly present on T and B lymphocytes wich has been used as part of conditioning regimens for prophylaxis against rejection and GvHD. In a group of patients with sickle cell disease undergoing matched sibling donor bone marrow transplantation, late alemtuzumab administration in the conditioning regimen (days −10 to −8) was associated with lower aGvHD but higher graft rejection compared to early alemtuzumab administration (days −19 to −17) (58). Several studies, mainly in reduced intensity conditioning HSCT for non-mailgnant diseases showed that alemtuzumab levels impact aGvHD, chimerism and lymphocyte recovery (59, 60), but also in malignant diseases (61). Although alemtuzumab abrogated severe GvHD this was not necessarily associated with improved OS (62). In a study of 148 patients comparing alemtuzumab and ATG, alemtuzumab delayed T and natural killer cell recovery compared with ATG and overall and event-free survival were lower in patients who received alemtuzumab. In addition, risk of recurrence of malignant disease was higher in patients who received alemtuzumab (63).

Abatacept is a recombinant fusion protein of CTLA4, a T-cell surface marker, and a fragment of immunoglobulin G. It intereferes with T cell priming and activation (55). Recently, a study in children and adults with haematologic malignancies undergoing HSCT from an unrelated donor matched at either 8/8 or 7/8 HLA-loci found that co-stimulation blockade with abatacept was safe and improved aGvHD rates: significantly fewer patients receiving a graft from a fully matched donor (8/8) and treated with abatacept as add-on to calcineurin inhibitor/methotrexate prophylaxis developed aGvHD (grade 2–4) when compared with the randomly assigned placebo group (43.1 vs. 62.1%, *p* = 0.006), with a trend toward decreased severe (grade 3–4) aGvHD. Patients receiving a partially matched graft (7/8) also demonstrated a sizable aGvHD benefit when compared with a matched control group receiving calcineurin inhibitor/methotrexaten only drawn from the Centre for International Blood and Marrow Transplant Research (CIBMTR) registry (2.3 vs. 30.2%, *p* < 0.001, for grade 3–4 aGvHD) (56). The additional immunosuppression by abatacept was not associated with an increased rate of relapse or infectious complications.

Post-transplant cyclophosphamide (PTCy) eliminates proliferating T cells and intra-thymic clonal alloreactive T-cell precursors while sparing regulatory T cells. PTCy-based GvHD prophylaxis has been a major advance allowing the widespread use of haploidentical HSCT; it is also gaining importance in HLA-matched and mismatched HSCT (57). Data in adults indicate that rates of severe aGvHD and cGvHD in the haploidentical HSCT setting are low with PTCy use (58). The situation in children seems distinct: haploidentical HSCT with use of PTCy has been associated with low rates of GvHD and NRM but delayed immune reconstitution, which might lead to a higher risk of infectious complications. Furthermore, whether the GvL effect (which is reflected by relapse rates), is maintained remains an important area of investigation (59). A detailed review regarding the use of PTCy vs. *in vitro* T-cell depletion (TCD) is provided by Kleinschmidt and colleagues in another review in this research topic section.

Graft engineering by various *ex vivo* TCD methods aims at maintaining anti-viral and anti-leukaemia activity while reducing the risk for GvHD. Such metods are: (1) the positive selection of CD34^+^ cells with or without a T-cell add-back at a later time point, or (2) the negative selection against CD3^+^ and CD19^+^ cells or (3) selective TCR αß^+^ and CD19^+^ depletion with preservation of γδ T cells and natural killer (NK) cells as well as (4) depletion of naïve CD45RA^+^ T-cells (60, 61). Di Ianni et al. evaluated a protocol with regulatory T-cell infusion 4 days prior to haploidentical transplantation in adult patients (*n* = 28) with haematological malignancies using CD34^+^ purified stem cells and add back of conventional T cells (up to 10^6^/kg). Immune recovery seemed enhanced compared to the standard haploidentical setting and the incidence of GvHD was low when the dose of conventional haploidentical T cells was limited to 10^6^/kg. Nonetheless, in this small, highly selected patient group, the rate of NRM was very high (50%). These deaths, mostly early post-transplant, were due to either regiment-related toxicity or infectious complications, indicating that despite accelerated immune recovery, this type of immune engineering still requires proof of clinical benefit (30, 62). Another cell type with immunomodulatory activity are invariant natural killer T cells (iNKT), which secrete interleukin (IL)-4 and IL-10(63). While still in its early phase, RGI-2001, a liposomal formulation of an alpha-galactosylceramide, has been shown to be taken up by dendritic cells, leading to iNKT activation and subsequent Treg expansion. A phase 1 (Clinicaltrials.gov NCT01379209) and a phase 2A study documented tolerability of the immune modulator and relevant expansion of regulatory T-cells in some patients (64).

## Clinical Staging and Grading of AGvHD

Early diagnosis and grading of aGvHD is essential to start therapy early in order to avoid the occurrence of a self-perpetuating inflammatory cycle. The first classification of aGvHD—based on clinical symptoms involving the skin, liver and gastrointestinal tract—was developed in 1974 by Glucksberg et al. (65). Later, the Keystone aGvHD Consensus Panel reviewed the outcome of the Glucksberg classification and confirmed the predictive value of maximum aGvHD grade for day +100 mortality (66). The International Blood and Marrow Transplant Registry (IBMTR) Severity Index tried to reclassify patterns of organ involvement into groups to make the index more sensitive and specific for studying aGvHD (67). The refined aGvHD Risk Score developed by the University of Minnesota helped to classify patients into standard and high-risk groups based on the clinical staging of the different affected organs (68). Harris et al. as part of the Mount Sinai Acute GvHD International Consortium (MAGIC), suggested some modifications to the Glucksberg scale, e.g., a standardised way to estimate and report stool output in children, including incorporating the number of episodes per day when quantification is not feasible (69) and this approach has been adopted in recent clinical trials. An EBMT–National Institutes for Health (NIH)–CIBMTR Task Force position statement details the different staging criteria, advocating for a standardised assessment of GvHD (70). Tables 1, 2 show individual organ severity staging and overall severity grading, respectively, according to the different classifications.

**Table 1:** Assessment of Acute GvHD: staging of severity for individual organs according to the different classifications.

**Organ**	**Severity stage**	**Modified Glucksberg (Keystone aGvHD consensus) criteria and IBMTR criteria (66, 67)**	**MAGIC criteria (68)**
Skin	0	No rash	
	1	Rash <25% BSA	
	2	Rash 25–50% BSA	
	3	Rash >50% BSA	
	4	Generalised erythroderma with bullous formation	Generalised erythroderma (>50% BSA) plus bullous formation and desquamation >5% BSA
Liver	0	Total serum bilirubin <2 mg/dL	
	1	Total serum bilirubin 2–3 mg/dL	
	2	Total serum bilirubin 3.1–6 mg/dL	
	3	Total serum bilirubin 6.1–15 mg/dL	
	4	Total serum bilirubin >15 mg/dL	
Upper GI tract	0	No persistent nausea and no histologic evidence of GvHD in the stomach or duodenum	No or intermittent anorexia or nausea or vomiting^*^
	1	Persistent nausea with histologic evidence of GvHD in the stomach or duodenum	Persistent anorexia or nausea or vomiting^*^
Lower GI tract	0	Diarrhoea ≤ 500 mL/day	Diarrhoea <10 mL/kg/day or <4 episodes/day^†^
	1	Diarrhoea >500 mL/day	Diarrhoea 10–19.9 mL/kg or 4–6 episodes/day^†^
	2	Diarrhoea >1,000 mL/day	Diarrhoea 20–30 mL/kg/day or 7–10 episodes/day^†^
	3	Diarrhoea >1,500 mL/day	Diarrhoea >30 mL/kg/day or >10 episodes/day^†^
	4	Severe abdominal pain with or without ileus	Severe abdominal pain with or without ileus or grossly bloody stools (regardless of stool volume)

*Adapted with permission from Schoemans et al. (70)*.

* *Anorexia accompanied by weight loss, nausea lasting ≥3 days or accompanied by ≥2 vomiting episodes per day for ≥2 days*.

† *One episode of diarrhoea in children weighing <50 kg is considered equivalent to 3 mL/kg. aGvHD, acute graft versus host disease; BSA, body surface area; GI, gastrointestinal; IBMTR, International blood and marrow transplant registry; MAGIC, Mount sinai acute GvHD international consortium*.

**Table 2:** Assessment of aGvHD assessment: overall severity grading according to the different classifications.

**Overall grade (modified glucksberg/MAGIC/Minnesota)**	**Modified glucksberg criteria (keystone aGvHD consensus) (66)**	**MAGIC criteria (69)**	**Minnesota criteria (68)**	**IBMTR criteria (67)**	**Overall grade (IBMTR)**
0	No organ involvement				0
I	Skin stage 1 or 2, without liver/GI involvement				A
II	Skin stage 3, and/or liver stage 1, and/or GI stage 1			Skin stage 2, and/or liver stage 1 or 2, and/or GI stage 1 or 2	B
III	Liver stage 2 or 3, and/or GI stage 2–4	Liver stage 2 or 3, and/or GI stage 2 or 3	Liver stage 2–4, and/or GI stage 2 or 3	Skin stage 3, and/or liver stage 3, and/or GI stage 3	C
IV	Skin stage 4, and/or liver stage 4	Skin stage 4, and/or liver stage 4, and/or GI stage 4	Skin stage 4, and/or GI stage 4	Skin stage 4, and/or liver stage 4, and/or GI stage 4	D

*Adapted with permission from Schoemans et al. (70)*.

*aGvHD, acute graft versus host disease; GI, gastrointestinal; IBMTR, International blood and marrow transplant registry; MAGIC, Mount sinai acute GvHD international consortium*.

## The Role of GvHD Biomarkers in Early Diagnosis, Risk Stratification and Response Assessment

Several markers of systemic inflammation such as IL-2Rα and tumour necrosis factor receptor 1 (TNFR-1) are correlated with GvHD outcomes (71). Markers of specific tissue damage have also been identified (72): elafin is specific for skin GvHD (73, 74), while hepatocyte growth factor is correlated with gastrointestinal and liver GvHD (75). Gastrointestinal GvHD is the major driver of aGvHD-related mortality (76). Suppressor of tumorigenesis 2 (ST2), which is shed from gastrointestinal tissue during GvHD, is a marker of GvHD treatment resistance and mortality (72). Regenerating islet-derived 3 alpha (REG3α) is released from Paneth cells into the circulation as a result of intestinal crypt damage (77).

Out of the different marker combinations, Reg3α and ST2 serum levels at the onset of GvHD, combined in an algorithm validated by the Mount Sinai Acute GvHD International Consortium (MAGIC), have been shown to be predictive of 6-month NRM independently of clinical severity at onset (78). Stratification is solely based on biomarker levels at the onset of GvHD, regardless of clinical severity; three risk categories (Ann Arbour 1–3) correlate with NRM. For instance, sometimes patients may present with relatively mild symptoms only to subsequently escalate to severe GvHD—this can be predicted by assessing the biomarkers in the serum. Vice versa, low biomarker levels at onset, even when clinical symptoms are severe, indicate a better chance for safe resolution of GvHD. Thus, a treatment approach guided by the MAGIC algorithm probability (MAP) might better identify patients in need of early escalation vs. those patients who will tolerate a rapid taper of immunosuppression.

While the MAP has been most extensively validated at the time of GvHD onset, identifying three risk categories, biomarkers have also been evaluated to assess response to treatment by day 28 from treatment initiation, indicating long-term outcome. Non-responders, assessed based on clinical symptoms only, are considered to have a 50% risk of NRM (68). Biomarkers allow a more refined analysis. At day 28 of treatment when using a single threshold validated for NRM, MAP can separate patients into high- or low-risk cohorts that are more predictive than the clinical response itself: in a prospective, multi-centre study, clinical responders with high biomarkers at day 28 had an NRM rate of 40% but those with low biomarkers had an NRM rate of 12%. Moreover, clinical non-responders with a low MAP had an NRM rate of 25% as opposed to 65% in non-responders with high MAP (79). The biomarker algorithm was recently validated in a paediatric cohort, with similar performance at onset and at day 28 (80).

Day 7 post treatment initiation is also a pivotal time point in the decision making for GvHD management, when GvHD is designated as treatment sensitive or refractory, decisions regarding escalation of therapy are made, and MAP helps to separate patients into high- and low-risk cohorts (78). For haematologic malignancies, especially high-risk leukaemias, identifying patients with a low risk of GvHD can be key to accelerating the tapering of immunosuppression and, thereby, possibly preventing early relapse.

## Differential Diagnosis to AGvHD

When aGvHD is suspected, it is important to rule out aetiologies other than GvHD that might exacerbate GvHD symptoms or require different treatment.

### Skin Rash

A rash a few days after conditioning is likely to be caused by TBI (especially at myeloablative doses of TBI used in ALL) (81) or chemo-conditioning. Thiotepa, melphalan, and etoposide can all cause skin toxicity. Moreover, exanthema due to ATG-based conditioning occurs frequently. The timing of occurrence usually helps to rule out aGvHD, as hyper-acute GvHD within the first 10 days after transplantation is considered a very rare event (69). Skin rash and pruritus can be seen during engraftment syndrome, a poorly defined immunological reaction occurring around the time of engraftment (82, 83). Vasculitis, hypersensitivity, drug reactions, and rashes caused by viral re-activation or dermatomycosis should also be ruled out.

### Colitis

Children with acute lymphoblastic leukaemia treated intensively before transplantation may carry multidrug-resistent bacteria in the gut that could affect the development of post-transplant intestinal GvHD (84). Since gut GvHD is the main reason for TRM following HSCT, and diarrhoea is the major clinical symptom used to stage gut GvHD, transplant physicians need to be very focused on this clinical parameter. However, alternative causes of gastrointestinal symptoms are viruses (adenovirus, cytomegalovirus, and norovirus) or pseudomembranous colitis due to *Clostridium difficile* toxin. MMF may cause gastrointestinal side effects including nausea (29%), vomiting (23%), constipation (38%), diarrhoea (50–92%), and colitis (9%) (85). In 98% of cases, resolution of diarrhoea occurs within 20 days upon discontinuation of the MMF (86). However, mycophenolate-mofetil–induced colitis is particularly challenging, as it requires a change in the immunosuppressive regimen rather than an increase in immunosuppression and is difficult to distinguish from GvHD even by histopathology (87), although important to assess for the differential diagnosis (88). In the majority of cases, patient's symptoms improve after lowering the dose or discontinuing the medication. Confirmation by biopsy of lower gastrointestinal GvHD is common practise and can help to rule out other aetiologies.

### Elevated Liver Enzymes

Liver GvHD is defined and staged by an increase of bilirubin (69). Elevation of liver transaminases may be associated with GvHD but is not a diagnostic criterion alone, although atypical hepatic forms of GvHD exist and may only be diagnosed by biopsy. Viral reactivation (adenovirus, cytomegalovirus, Epstein-Barr virus) and drug-related toxicity cause liver enzyme elevation much more frequently than GvHD, as the incidence of liver GvHD after HSCT is low. Furthermore, liver toxicity related to previous ALL treatment is frequent and therefore patients may present to HSCT already with elevated liver enzymes (89, 90). Isolated liver GvHD is even more rare, whereas liver GvHD in combination with severe gut GvHD is a more common clinical picture. Veno-occlusive disease, which also can cause elevated bilirubin levels, is an important differential diagnosis as its management differs greatly from GvHD and it may be life-threatening when unchecked.

### Inflammation

Sub-febrile temperatures and slightly elevated levels of C reactive protein are seen frequently post HSCT. While transplant physicians are trained to rule out ongoing infection (by bacteria, viruses, *Aspergillus*, and *Candida*), there are no defined markers that would allow clinicians to discern milder infections from a merely inflammatory reaction of the newly established immune cells that does not meet the criteria for aGvHD. When inflammation occurs at the time of engraftment, such an inflammatory reaction may be termed “engraftment syndrome,” although a potential overlap with GvHD may exist (82, 83). Finally, in malignant diseases, relapse of the underlying disease should be ruled out if inflammatory signs or symptoms persist.

## Acute GvHD Treatment

In general, treatment for aGvHD should aim for resolution of manifestations yet limited treatment-related toxicities. This goal should be achieved at the lowest cost of cure to maintain the GvL effect and to minimise the impact on immune reconstitution and infectious complications.

### First-Line Treatment

The first-line treatment approach is summarised in Table 3. Corticosteroids are the mainstay of GvHD therapy (91, 92). Their effects are complex and not completely understood. However, it has been shown that a main mechanism of steroids in aGvHD is the inhibition of nuclear factor κB pathways in antigen-presenting cells and T cells as well as inhibition of Toll-like receptor (TLR) signalling (93–95). In T cells, steroids suppress activation and proliferation (96, 97) and reduce the production of chemokines and expression of adhesion molecules in a manner that decreases the migration of donor T cells into target tissues (98).

**Table 3:** Suggested first-line treatment of aGvHD in children.

**Grade**	**Treatment**
Grade I aGvHD	Topical treatment with either steroids or calcineurin inhibitor (tacrolimus or pimecrolimus)^*^. In younger children, side effects may occur more frequently due to the larger ratio of skin surface to body weight. Potent steroids should not be applied to the face
Grade II aGvHD with isolated skin or upper gastrointestinal tract^†^	Low-dose steroids: 1 mg/kg/day prednisone or methylprednisolone plus topical treatment (Combine with topical treatment)
Grade III aGvHD beyond isolated skin or upper gastrointestinal tract	Steroids: 2 mg/kg/day prednisone or methylprednisolone. For patients with gastrointestinal involvement or oral intake impairment, the intravenous route would be of choice (Combine with topical treatment)
Grade III and IV aGvHD	Steroids: 2 mg/kg/day prednisone or methylprednisolone. For patients with gastrointestinal involvement or oral intake impairment, the intravenous route would be of choice (Combine with topical treatment)

* *Topical treatment to relieve itching and prevent skin breakdown: (1) hydrocortisone 0.5–1% when the skin involvement is very superficial and also for delicate areas, such as the face and genital area, (2) betamethasone and triamcinolone in the case of more intense affectation but avoiding use in delicate areas; or (3) a topical calcineurin inhibitor (tacrolimus and pimecrolimus)*.

† *Gastrointestinal aGvHD may benefit from topical steroids in a non-absorbable form, i.e., beclomethasone or budesonide. aGvHD, acute graft versus host disease*.

There is high inter-centre variability in the starting dose of steroids, with the majority of physicians favouring lower doses of prednisone and methylprednisolone in mild-to-moderate GvHD. The initiation of systemic steroids should be based on organ involvement and GvHD stage/grade (69): patients should receive the lowest effective dose of prednisone or methylprednisolone in order to reduce the risk of side effects (99–101). Topical application of steroids for skin GvHD and use of poorly absorbable corticosteroids with high first pass for gut GvHD should be taken in consideration early on to reduce the use of systemic steroids where appropriate (102, 103). Co-medication with calcineurin inhibitors should be maintained while on therapy, with trough levels for cyclosporine A adjusted to higher levels (200–300 ng/mL) if tolerated (104).

Tapering corticosteroids is generally highly individualised based on each patient's risk factors as well as depending on each centre's standard procedures. Steroids are essential in most ALL treatment protocols and this is why these patients, even before transplantation, already may have complications due to steroid use (105). As a general rule, one should aim for the lowest effective dose of corticosteroids for the shortest period possible. In the absence of clinical signs, most paediatric centres normally taper prednisone doses by 20–25% every 3–7 days, depending on response (50). However, the withdrawal of steroids must be carried out carefully, since during the steroid taper there may be a reappearance of GvHD. The MAGIC biomarker algorithm at day 7 and 28 post initiation of steroids may serve to assess response and help to guide steroid taper (78, 79).

### Steroid-Refractory GvHD

Only 30–50% of children respond to corticosteroids as initial therapy for GvHD. Similar to the heterogenous activity of GvHD, there are many possible scenarios where steroids might not work, as reviewed extensively by Toubai and Maganeu (98). There may even be paradoxical effects due to disturbance of the balance of lymphocyte subsets, leading to a dominance of the pathological IL-2- and IL-17-producing T-helper cell response or the perpetuation of TLR/NLRP3 expression with maintained inflammation. Furthermore, steroids impede rather than support reparative processes such as the re-building of the gut mucosa (96).

The definition of steroid-refractory GvHD has been difficult to establish. While the EBMT European LeukemiaNet recommendation is the diagnosis of steroid-refractory GvHD after 5 days of non-response to steroids (46, 106), most paediatrics groups consider patients to be steroid refractory after a shorter period of time, diagnosing first-line treatment failure after 3 days if any organ progression occurs (50). The early diagnosis of steroid-refractory GvHD in paediatric patients allows the early introduction of second-line therapies. Table 4 describes the EBMT-NIH-CIBMTR criteria for defining steroid-refractory, -dependent and -intolerant aGvHD.

**Table 4:** EBMT-NIH-CIBMTR criteria to define steroid-refractory, -dependent, and -intolerant aGvHD.

**Terminology**	**Definition(s)**
Steroid-Refractory aGvHD	– Progression of aGvHD within 3–5 days of therapy onset with ≥2 mg/kg/day of prednisone – Failure to improve within 5–7 days of treatment initiation with ≥2 mg/kg/day of prednisone – Incomplete response after > 28 days of treatment with ≥2 mg/kg/day of prednisone
Steroid-Dependent aGvHD	– Inability to taper prednisone <2 mg/kg/day after an initially successful treatment of ≥7 days – Recurrence of aGvHD activity during steroid taper
Steroid-Intolerant aGvHD	– Occurrence of unacceptable toxicity due to the use of corticosteroids

*Adapted with permission from Schoemans et al. (70)*.

*aGvHD, acute graft versus host disease; EBMT, European society for bone and marrow transplantation; CIBMTR, Centre for international blood and marrow transplant research; NIH, National institutes for health (US)*.

### Second-Line Treatment

#### Conventional Pharmacological Intervention

Second-line treatment for GvHD is recommended if refractoriness, dependence or intolerance to steroids is established. Given the generally severe clinical picture of GvHD, second-line therapy is usually added on top of the existing therapy regimen or begun in an overlapping schedule. However, in a survey on current practise in 75 paediatric centres, the majority (92%) indicated that they would stop giving steroids once an alternative therapy was established (50).

Table 5 summarises the published studies of second-line treatments for GvHD in children, including response rates, and toxicities (107–125).

**Table 5:** Studies of conventional pharmacological second-line treatments for steroid-refractory aGvHD that included children.

**Investigational agent**	**Study design**	**Patients, *N*/years**	**Overall response rate**	**Complete response rate**	**Overall survival rate**	**Main toxicities**	**References**
Ruxolitinib	Phase III	154/12–73	62%	40% (durable response on day 56)	53% at 1 year	Thrombocytopenia: 33% Anaemia: 30% CMV infection/reactivation: 39%	Zeiser et al. (107)
Mofetil mycophenolate	Phase II	26 (13with aGvHD)/17-53	31%	15%	33% at 2 years	For the whole population: Gastrointestinal: 27% Infection: 31% CMV: 11%	Kim et al. (108)
	Phase II	19/4–54	47%	31%	16% at 1 year	Neutropenia:10.5% Abdominal pain: 5% Pulmonary infiltrates: 5% Neutropenia+gastrointestinal toxicity: 15,8% Infection as cause of death: 32%	Furlong et al. (109)
	Retrospective	14/0–17	79%	50%	85%, median follow-up 35 months	CMV reactivation: 29% CMV retinitis: 7% ADV infection: 7% Haemorrhagic cystitis: 14% Aspergillosis: 7% Neutropenia: 7% Thrombocytopenia: 7%	Inagaki et al. (110)
Anti-TNF antibody infliximab	Retrospective	24 (22 assessable for response)/0–18	82%	54%	46% at 1 year; 21% at 2 years	Bacterial infection: 77% Viral infection: 32% Fungal infection: 13.6%	Sleight et al. (111)
	Retrospective	32/2–66	59%	19%	41%	Infections in 72% Septicaemia and septic shock: 22% Pneumonia: 28% Enteritis: 12.5% Encephalitis: 3% CMV reactivation: 41% Invasive fungal infection: 6%	Patriarca et al. (112)
ATG	Retrospective	79/NA	54%	20%	32% at 1 year	Bacterial infection: 37% Fungal infection: 18% CMV: 10%	MacMillan et al. (113)
	Phase II/III	ABX-CBL: 48/2–65; Horse ATG: 47/2–65	ABX-CBL:56%; ATG:57%	ABX-CBL:29%; ATG:32%	ABX-CBL:35% at 18 months; ATG: 45% at 18 months	Infections: 98% (ABX-CBL), 100% (ATG) Fever: ABX-CBL 20%, ATG 30% Hypertension: ABX-CBL 30%, ATG 28% Hyperglycaemia: ABX-CBL 24%, ATG 26% Abdominal pain: ABX-CBL 15%, ATG 33%	MacMillan et al. (114)
Alemtuzumab	Retrospective	18/1–59	83%	33%	55% at 11 months	Infection: 78% CMV reactivation: 67% Grade 3 neutropenia: 33% Grade 3 thrombocytopenia: 22% Chills, fever and headache: 28% Tuberculosis: 1 patient	Gomez-Almaguer et al. (115)
	Phase II	18/13–68	99%	28%	33% at 36.5 weeks	Sepsis: 28% Pneumonia: 39% Viral infection: 44% Fungal infection: 22% CMV: 56% EBV: 11%	Schub et al. (116)
	Phase I/II	15/1.4–27	67%	40%	80% at 6 months	Fever: 26% Thrombocytopenia: 53% Viremia: 100% CMV disease: 2 patients EBV PTLD: 1 patient	Khandewall et al. (117)
Anti IL-2 receptor antibody daclizumab	Retrospective	13/paediatric	92%	46%	46% at 14 months	CMV reactivation: 54% VVZ reactivation: 15% Sepsis: 8% EBV reactivation15%	Miano et al. (118)
	Phase II	62/1–53	90%	68,8%	54.6% at 4 years	CMV reactivation: 39% Infections as cause of death: 11%	Bordigoni et al. (119)
	Retrospective	57/0–57	54%	76% for patients ≤ 18 years old	Median survival: 3.6 months	Opportunistic infection: 95% Bacterial infection: 88% Fungal infection: 51% Viral infection: 53% CMV: 35% EBV: 7%	Perales et al. (120)
Anti IL-2 receptor antibody basiliximab	Retrospective	34/2–38	82%	32%	20% at 5 years	NA	Funke et al. (121)
	Retrospective	230 (74 <18 years)	78.7	60.9	61.7% at 4 years	Bacterial infection: 52.6% Fungal infection: 16.1% Viral infection: 3.8%	Liu et al. (122)
	Retrospective (haploidentical HSCT)	100/1–17	85%	74%	76,2% at 3 years	Bacterial infection: 11% Fungal infection: 7% CMV viremia: 53% EBV viremia: 11% HHV-6 viremia: 7%	Tang et al. (123)
Basiliximab + etanercept	Prospective	65/9–55	90.8%	75.4%	54.7% at 2 years	Cytopenia: 49.2% Haemorrhagic cystitis: 28% Fungal infection: 36% CMV reactivation: 57% EBV reactivation: 6.2%	Tan et al. (124)
Pentostatin	Phase I	23(22 assessable for response)/0–63	77%	64%	26%, median survival 85 days	Lymphopenia: 100% Thrombocytopenia: 4% Infection: 9%	Bolaños-Meade et al. (125)

*aGvHD, acute graft versus host disease; ATG, anti-thymocyte globulin; CMV, cytomegalovirus; EBV, Epstein-Barr virus; HSCT, haematopoietic stem cell transplantation; HHV-6, human herpesvirus 6; IL, interleukin; PTLD, post-transplant lymphoproliferative disorder; TNF, tumour necrosis factor*.

Despite a multitude of Phase I/II studies conducted over recent decades assessing a range of different compounds, there were no drugs approved for the treatment of steroid-refractory aGvHD for adults or children until recently. In 2019, new data including from a Phase III clinical trial led to the approval of ruxolitinib for the treatment of steroid-refractory aGvHD in children ≥12 years by the US Food and Drug Administration (107, 126, 127). Ruxolitinib is a selective inhibitor of Janus kinase 1/2, thus it targets a central pathway in the pathogenesis of GvHD. The Janus kinase pathway is crucial for the release of inflammatory cytokines and subsequent activation of antigen-presenting cells, which affects the priming, and activation of alloreactive T cells as well as their migration and cytotoxic activation. In addition to interfering with this cycle of activation, ruxolitinib boosts the proportion of regulatory T cells in relation to conventional CD4^+^ T cells. Importantly, experiments from mouse models of aGvHD indicate that the GvL effect of HSCT is preserved with ruxolitinib use (128), which is an important issue especially in patients with malignant disease.

The prospective, multicentre, Phase II REACH 1 trial showed that, at day 28 post initiation of treatment, patients ≥12 years old with grade II–IV steroid-refractory or steroid-dependent aGvHD who received ruxolitinib in combination with steroids had an overall response rate of 55% and complete response rate of 27%. Of patients who had a complete response, the median duration of complete response was 1 year and overall survival was 51% at 6 months (127). These results led to the Phase III REACH 2 trial in which patients aged 12 years and older were randomised in a 1:1 ratio and received either ruxolitinib (10 mg twice daily) or the investigator's choice of therapy from a list of nine commonly used options. Three hundred and nine patients were treated. Median OS was 11 months for the ruxolitinib group and 6.5 months in the control group. The rate of overall response at day 28 was higher in the ruxolitinib group than in the control group [62 vs. 39%, respectively; odds ratio 2.64, 95% confidence interval (CI) 1.65–4.22; *p* < 0.001]. Durable overall response at day 56 was higher in the ruxolitinib group than in the control group (40 vs. 22%, respectively; odds ratio 2.38, 95% CI 1.43–3.99; *p* < 0.001). Thrombocytopenia was significantly more frequent in the ruxolitinib group than in the control group (107).

Retrospective studies have also analysed ruxolitinib use in children at the dose of 5 mg every 12 h for those ≥25 kg body weight and 2.5 mg every 12 h for those <25 kg. In a study of 13 patients (age 1–16 years) with steroid-refractory aGvHD of whom 11 were evaluable for response, five patients had an overall response, one had a complete response and two had no response. Four patients had treatment failure because of toxicity (129). In four more-recent studies, better overall response rates of 77–84% and complete response rates of 31–69% were reported (130–133). Adverse events included grade 3 thrombocytopenia and neutropenia, infectious complications, and Epstein-Barr virus post-transplant lymphoproliferative disease (130). Final results of the REACH 4 trial—a prospective, multicentre, Phase II clinical trial of ruxolitinib for either steroid-refractory aGvHD or as add on to steroids at aGvHD onset in children aged 0 to <18 years of age are awaited.

It is clear that, even with more data on ruxolitinib becoming available, some patients will not respond or cannot tolerate ruxolitinib. Thus, the need for an effective treatment strategy for steroid refractory aGvHD with limited toxicity remains high.

#### Extracorporeal Photopheresis

Extracorporeal photopheresis (ECP) is a physicochemical procedure that induces apoptosis in collected mononuclear cells by extracorporeally sensitising them with 8-methoxypsoralen (8-MOP) and subsequently exposing them to ultra-violet A light. Although many aspects of ECP are not yet fully understood, the general principle appears to be the modulation of the antigen-presenting compartment to induce tolerance (134): after the re-infusion of these cells, apoptotic bodies are picked up by antigen-presenting cells, which, in consequence, down-regulate their inflammatory signature (reduced IL-2, tumour necrosis factor α, and interferon γ) and upregulate a more anti-inflammatory profile (tumour growth factor β, IL-10). This leads to reduced T-cell stimulation, an increase in regulatory T cells and, at best, tolerance induction (135–137).

Technically, there are three options for performing ECP. In the “off-line” system (known also as the open system), the leukapheresis product is collected first. In a separate step, the cells are then treated with 8-MOP and exposed to ultra-violet A light, followed by re-infusion into the patient. In the “in-line” system (known also as the closed system) those two processes are integrated in one machine, while using a discontinuous flow cell separator (138). The US Food and Drug Agency and European Medicines Agency approved this later technique for the treatment of steroid-refractory aGvHD and cGvHD. Both processes require good venous access to allow continuous blood flow during leukapheresis. This may prove difficult in many patients, especially as repetitive treatments are needed. In these situations, a third option—so-called mini-ECP—may be used. Mini-ECP uses the white blood cells from the buffy coat prepared from whole blood (5–8 mL/kg), that is collected, treated and reinfused in a closed system. While fewer cells can be collected at a given time, the number of collected cells required to induce tolerance can be reached for small children (139, 140).

Even though ECP is well-tolerated in children, leukapheresis procedures are technically challenging. ECP in children differs from ECP in adults because of the distinct physiological features of children and, thus, requires clinicians to have specialised knowledge and experience to perform it safely, especially in low-weight children (141). Major concerns are: (1) the significant fluid shifts that occur during leukapheresis potentially resulting in haemodynamic instability; (2) achieving vascular access with sufficient flow rate; (3) haematologic and metabolic disturbances; and (4) the duration of leukapheresis procedures, often necessitating the sedation of infants and toddlers.

The ECP treatment schedule varies depending on aGvHD activity but 2–3 sessions per week are considered necessary in the initial induction period (142, 143). Although ECP is seen as a second-line strategy for steroid-refractory aGvHD in the paediatric setting, data from randomised clinical trials in adults and/or children are scarce. Evidence is mostly based on case reports, case series or observational studies, where response rates range from 50 to 100% depending on organ involvement (144, 145). In two adult patient cohorts with a total of 59 patients with steroid-refractory or steroid-dependent aGvHD, Greinix et al. report high response rates to ECP. Most notably, an early start of treatment and treatment intensification led to a 43–60% response rate in grade III/IV steroid-refractory GvHD (146). A prospective, international trial (Clinicaltrials.gov identifier NCT02524847) including 29 children has recently closed but the results were not published at the time of writing (CDH, personal communication).

Given that ECP is time intensive, identifying early on with biomarkers those patients who are likely to respond or not respond to this therapy would be extremely beneficial. Pilot studies on small numbers of patients focusing on changes in the T-cell (147) and NK-cell (148) compartment suggest favourable shifts toward a more tolerant immune cell signature. Whether such signatures or established biomarkers will help to discern between likely refractory vs. responding patients and better define the patient population that benefits from ECP requires further evaluation.

#### Mesenchymal Stromal Cells

Mesenchymal stromal cells (MSCs) are multipotent non-haematopoietic stem cells originally isolated from bone marrow; they have multiple immunomodulating functions. Besides the bone marrow, MSCs they can be found in and grown from a variety of tissues including adipose tissue and umbilical cord (149, 150). In addition to their immunomodulatory potential, MSCs are thought to contribute to repair and regeneration of diseased or damaged tissue, especially in the state of severe endothelitis and small-vessel disease (151). As a “living pro-drug” the inflammatory signals within the host stimulate MSCs to counteract inflammation by secreting anti-inflammatory mediators before they quickly disintegrate.

A meta-analysis by Morata-Tarifa et al. analysed data from 51 mostly small studies (152). Across the combined population of adults and children, patients with steroid-refractory aGvHD receiving MSC were shown to have a survival advantage (878 patients, 50% alive at last follow-up) over a control group (pooled data from 5 studies: 182 patients, 25% alive at last follow-up). The most recent update from a study of the bone-marrow-derived MSC product “MSC-Frankfurt am Main” (MSC-FFM) in 92 patients (two-thirds of whom were children and adolescents) with severe steroid-refractory aGvHD reported an overall response rate of more than 80% and OS at 6 months of 64% with a median of three doses (range 1–9) of 0.6–4.5 × 10^6^ MSCs/kg administered at approximately 1-week intervals (153). A randomised Phase III trial in steroid-refractory aGvHD which is open to children was recruiting at the time of writing (Treatment Of Steroid-Refractory Acute Graft versus host Disease With Mesenchymal Stromal Cells Vs. Best Available Therapy (IDUNN) Clinicaltrials.gov identifier: NCT04629833).

An initial Phase III trial with remestemcel-L (*ex vivo* culture-expanded allogeneic adult human MSCs distinct from the IDUNN product) for steroid-refractory aGvHD included 163 patients aged 6 months to 70 years with steroid-refractory aGvHD who received MSC and 81 control patients (154). This trial showed that there was no significant difference in survival by day 180 between the two treatment arms but indicated a benefit of remestemcel-L for certain subgroups, such as paediatric patients. In a multicentre expanded-access protocol using remestemcel-L, 241 paediatric patients with steroid-refractory aGvHD, the majority of whom were resistant to multiple immunosuppressive therapy at the time of study enrolment, were treated with remestemcel-L as salvage therapy. The overall response rate at day 28 was 65% and responder survival at day 100 was significantly greater than non-responder survival (82 vs. 39%, respectively; log rank *p* < 0.001) (155). More recently a Phase III, single-arm, prospective study of remestemcel-L showed a day-28 overall response rate of 69.1%; 74.5 and 68.5% of patients were alive at days 100 and 180, respectively (156). Biomarker analysis for these patients showed that seven of 11 patients characterised as high-risk by MAP responded to MSCs and were alive at 6 months, comparing favourably to a control group, matching the clinical criteria but not having received MSCs, and having a similar MAP profile (157).

There are no known contraindications to MSCs and cross-reactivity of MSCs with most other relevant medicines has not been seen so far. Avoidance of prostaglandin synthesis inhibitors is recommended due to the partial dependence of anti-inflammatory effects on prostaglandin E2. All reports agree on the excellent safety of MSCs in aGvHD (155, 158, 159). However, given the variable nature of a cellular product, where many details of the production process might vary from study to study and even from batch to batch, and because of differences between the respective studies (e.g., GvHD stage and organ involvement), a definitive evaluation of who—if anyone—might benefit from this type of therapy is still pending.

#### Future Therapeutic Strategies

Table 6 summarises promising novel strategies in steroid-refractory aGvHD. Recently, vedolizumab—an antibody blocking α4β7 integrins—has demonstrated promising activity in aGvHD. As gut GvHD is the leading cause of TRM, the promotion of the migration of alloreactive T cells to the gut by the inhibition α4β7 integrins may be a useful strategy. The few published Phase I studies of vedolizumab in this setting showed some responders to vedolizumab but larger, Phase III studies are missing (164, 167–170). Intriguingly, Mehta et al. reported recently that six of 12 adult GvHD patients not responding to ruxolitinib benefitted from vedolizumab as third-line GvHD treatment (164). However, in view of the proposed mechanism of action of vedolizumab and given that upregulation of α4β7 integrins has been observed 1 week before onset of GvHD (167), the use of vedolizumab as early treatment rather than third-line therapy may be a central question for future clinical trials. Natalizumab, which acts against the α4 subunit that mediates homing of lymphocytes to the GI tract, was also evaluated in a Phase II study including 21 adults, demonstrating safety and durable responses in 6 of 8 CRs (165). Natalizumab is currently being evaluated by the MAGIC group with a biomarker-guided risk stratification (Clinicaltrials.gov Identifier: NCT02133924).

**Table 6:** Summary of novel and potential future strategies for the management of steroid-refractory aGvHD.

**Strategy**	**Pharmacological and non-pharmacological options**
Promote intestinal repair in patients with denuded mucosa	Lithium (160), glucagon-like peptide 2 (161), Visilizumab (IgG2 Fc) (162)
Reduce dysbiosis of the gut microbiome	Faecal microbiota transfer (163)
Modification of alloreactive T cells	Anti-Integrin α4β7 (vedolizumab) (164); Natalizumab (165)
JAK-1 inhibitor, cytokine blockade, combination therapy	Itacitinib + tocilizumab (anti IL-6 receptor antibody) (166)
Induce apoptosis of activated T lymphocytes	Neihulizumab (binds CD162) (Clinicaltrials.gov Identifier: NCT03327857)

*aGvHD, acute graft versus host disease; IL, interleukin; JAK-1, Janus kinase 1*.

Other strategies to selectively targeting alloreactive T cells have been approached. These T cells express CD30 and so might be targeted by brentuximab vedotin. Early results of a Phase 1 study of brentuximab vedotin in 34 adults with steroid-refractory GvHD showed responses in 38% of patients but TRM due to infectious complications was dose limiting (171). A retrospective study of the use of anti-IL-6 receptor antibody tocilizumab in 16 adults with biopsy-proven steroid-refractory gut aGvHD reported responses in 10 of the patients (62.5%) (166). Regeneration of host tissues may be of special interest in patients with steroid-refractory GvHD and profound tissue damage. Alpha 1 antitrypsin prevents organ damage by inhibiting neutrophil elastase and possesses immunomodulatory functions, suppressing proinflammatory cytokines and inducing regulatory T cells. In a phase 2 trial of alpha 1 antitrypsin including 40 adults with steroid-refractory aGvHD, a response rate of 65% and a relatively low rate of infectious mortality (4 patients) has been reported (172). In other studies, lithium was found to promote intestinal repair in patients with denuded mucosa (160) and IL-22 restored regenerating islet-derived protein 3 γ production lost after Paneth cell destruction and facilitated the regeneration of gut epithelium in HSCT models (173). Reducing dysbiosis of the gut microbiome may also help in the treatment of steroid-refractory GvHD (163, 174, 175).

The conduct of clinical trials in aGvHD is riddled with many challenges. In children, a significant obstacle is the low number of patients, making paediatric only GvHD trials difficult to complete. Despite the lower risk of developing GvHD in children, the poor outcomes of refractory GvHD, and validation of prognostic biomarkers support the inclusion of children in risk based trials.

## Supportive Treatment

Supportive care during paediatric HSCT has been recently outlined in other reviews (176, 177); therefore, only the most relevant issues to GvHD are summarised here.

Prophylaxes to prevent viral and fungal disease as well as *Pneumocystis jirovecii* infection are indicated for immunosuppressed patients following HSCT. Data on the potential benefits and disadvantages of gut decontamination to reduce gramme-negative entry into the bloodstream during the vulnerable phase following conditioning are not available. In light of a potentially protective role of a diverse microbiome against GvHD (178, 179), restricting antibiotics directed against anaerobic bacteria especially 1 week prior and 1 week after HSCT whenever possible may reduce the rate of acute gut/liver GvHD, but requires careful monitoring for infections (180). In adults undergoing HSCT, use of rifaximin for gut decontamination has been shown to better maintain the diversity of the gut microbiome than use of ciprofloxacin/metronidazole (181).

During the first weeks after HSCT, patients require various therapeutic and prophylactic drugs, e.g., antibiotics, virostatics, and immunosuppressive. Their side effects and potential interactions need to be closely monitored. Of particular concern, glucocorticoids can have detrimental long-term effects in children, including an increased the risk of infectious complications, hormonal, and growth disturbances and avascular necrosis of the bones. This latter condition carries a high burden of morbidity, as prolonged or even permanent functional impairment and chronic pain may occur. There is consensus on neither the risk factors for development of avascular necrosis in children receiving glucocorticoids nor the best strategies for prevention and treatment, which remain unmet clinical needs. However, calcium and vitamin D levels should be monitored and supplemented as necessary and the initiation of physiotherapy is recommended.

Nutritional disturbances, especially in patients affected by gastrointestinal aGvHD, can cause weight loss, malnutrition and atrophy of intestinal microvilli. Even with nausea, vomiting, or diarrhoea, providing nutrition via the gastrointestinal route is preferred and may be facilitated using a nasogastric tube if necessary. When the intestinal barrier is not intact or there is a malabsorption, hydrolysed formulas (which are also used successfully in auto-immune colitis) (182) or elemental formulas can be offered to patients. Whether or not a highly hydrolysed formula contributes to reduced inflammation in the context of GvHD is an interesting yet untested hypothesis. Some patients will require parenteral nutrition alone or in combination with the enteral nutrition. Supplementation with vitamins and trace elements may be applied in line with guidelines on parenteral nutrition, but data on the benefit of such supplementation are scarce (183).

## Discussion and Future Directions

It is indisputable that paediatric care in many ways requires a different approach than that used for adult medicine. ALL is a prime example, as permanent cure can now be achieved for >90% of our young patients (184). For those with a high-risk profile or relapse, HSCT is a key element to establish long-term control based on GvL activity. Results obtained in the FORUM trial showed a higher probability of EFS in patients experiencing grade II aGvHD than in patients with no signs or very mild GvHD (°I), suggested that—at a moderate stage—aGvHD is associated with a GvL effect and protects from leukaemia recurrence (1). A study conducted by the Children's Oncology Group showed decreased relapse risk in patients developing Grade 1–3 aGvHD in a multivariate analysis controlling for pretransplant MRD (15). Similarly, a combined analysis of several databases from North America, Europe and Australia, showed that in addition to post HSCT MRD, aGvHD significantly impacted risk of relapse, controlling for post HSCT MRD (16). Thus, while aGvHD of grade III and IV needs to be avoided due to its difficult course and potentially life-threatening consequences, the challenge lies in achieving and allowing sufficient alloreactivity to target residual leukaemic cells. Given the high potential of immunological recovery in children (e.g., supported by residual thymic function), we need to better understand the age-dependent control of the developing immune system. Immunologically, the early days post-transplant may have the most impact on how alloreactivity and subsequent GvHD are triggered, yet clinical symptoms follow only after a time delay. In ALL, TBI is effective for myeloablation prior to HSCT but it stresses non-haematopoietic cells to express co-stimulatory molecules and major histocompatibility complex class II (37), paving the way to stimulating alloreactive donor T cells. In this regard, one can speculate, that TBI allows for a better GvL effect than chemo-based conditioning, although the FORUM trial was not designed to answer this specific question. Tailoring GVHD prophylaxis to donor type, to maintain GVL, without increasing severe GVHD, however, is challenging. Choosing the appropriate donor is a key question as it directly affects EFS, GvHD and NRM. It is beyond the scope of this review to comprehensively discuss donor selection, but ideally GvHD prophylaxis is adapted according to the type of donor.

Appropriate timely withdrawal of immune suppression in the absence of GVHD, and tapering immunosuppression once a GVHD response is achieved, is critical. In the study conducted by the Children's Oncology Group Pulsipher et al. showed that patients with ALL who were MRD+ pre-transplant and developed aGvHD in the first 2 months after HSCT did not relapse. Consequently, patients who do not develop aGvHD in the first 2 months are candidates for rapid withdrawal of immunosupression (20). It has also been shown that rapid withdrawal of immunosupression can be safely performed in high-risk population with important improvement in survival (21). Assessing relapse in GVHD prophylaxis and treatment trials is essential.

Also, biomarkers for early detection of GvHD can help the clinician to be one step ahead in the management of GvHD, but controlled clinical studies are required to ensure that biomarker-guided, pre-emptive immunosuppressive therapy benefits the patient without leading to high rates of overtreatment. Patients at low-risk of GvHD can be identified more easily by measuring serum markers, especially ST-2 and Reg3a. When using the MAGIC algorithm, such low-risk patients could be tapered off immunosuppression more rapidly than patients with a higher MAP, and this will be tested in an upcoming paediatric clinical trial within MAGIC centres. Rapid, but safe taper might help prevent leukaemic relapse.

Guidelines to standardise the clinical staging of GvHD are now widely accepted, but harmonisation between centres still requires a high level of exchange and communication. Unified classification via an electronic scoring App (similar to the educational EBMT GvHD App developed for adults; https://www.ebmt.org/education/apps) should be a goal in the paediatric setting.

In adults, several recommendations for aGvHD management from different scientific societies have been published (46, 185, 186). In these guidelines, there is a consensus on the importance of promoting the treatment of patients in clinical trials in order to elucidate better strategies for the management of steroid-refractory aGvHD. In children, the scarcity of data is even greater and, therefore, the inclusion of children in clinical trials of biomarker-guided early treatment interventions to decrease NRM and toxicity is of the utmost importance (91). Ideally, such trials would be designed and powered in a way that specific insights can be gained for this vulnerable, young patient population. As observed in daily clinical practise, one particular treatment may not fit all patients, underlining the importance of a personalised strategy according to individual characteristics. For example, active infection, a history of thrombotic events or persistently low platelet counts will influence the physician's decision of whether to use second-line drugs (such as ruxolitinib or antibodies) or alternative treatment options such as ECP or MSC.

Traditionally, treatment of steroid-refractory aGvHD has focussed on the intensification of immunosuppression; however, as more knowledge on the immunopathology of GvHD has been gained, more selective treatments have become available, such as targeting alloreactive T cells or the use of anti-cytokine antibodies (187). Furthermore, other mechanisms might contribute to steroid-refractory aGvHD and be approached by other non-immunosuppressive treatments (188). For example, as impaired epithelial regeneration is described in gastrointestinal GvHD and for patients with denuded intestinal mucosa, new ways to promote intestinal repair are needed rather than just adding immunosuppression (189). Alterations in the composition of intestinal microbiota may drive persistence of the disease in some patients and many investigations are ongoing to address this issue (163, 174, 190). Severe aGvHD can also cause endothelial injury resulting in thrombotic microangiopathy. This course of the disease then requires a different management, as immunosuppression alone is likely to be insufficient (191).

**Figure 1:**
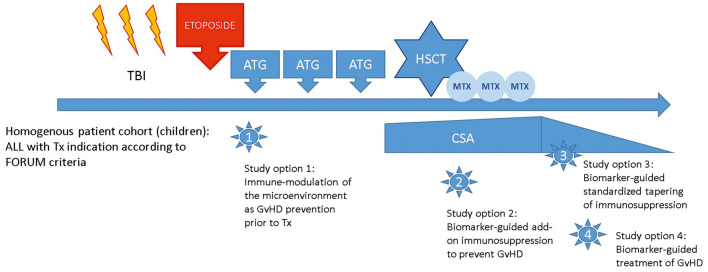
Study options building on the PED-FORUM experience.

For paediatric ALL patients with an indication for HSCT, the FORUM trial confirmed TBI to be the current standard of care for conditioning in patients with ALL aged 4 years or older (1). These patients are a uniquely homogenous patient population with a uniform diagnosis of high-risk ALL transplanted according to a standardised conditioning regimen. Based on the experience of the FORUM trial, the network is well-suited to tackle further research questions regarding the prevention and management of GvHD while maintaining the GvL effect in these children as illustrated in Figure 1.

## Author Contributions

MW provided content of critical importance, drafted the manuscript, and approved the final version. MQ, MB, TS, HB, and AL provided content of critical importance, revised the manuscript, and approved the final version. CD-d-H coordinated the writing process, provided content and structure of critical importance, revised the manuscript, and approved the final version. All authors contributed to the article and approved the submitted version.

## Funding

This study received funding from the St. Anna Children's Cancer Research Institute, Vienna, Austria. The funders were not involved in the study design, collection, analysis, interpretation of data, the writing of this article, or the decision to submit it for publication.

## Conflict of Interest

CD-d-H has acted as a consultant and speaker for and has received travel expenses from Novartis. MW has acted as a consultant for and has received travel expenses from Novartis. MW also received travel expenses from Mallinckrodt Pharmaceuticals. MQ received honoraria from Mesoblasts, Medexus, Jazz Pharmaceuticals, and Novartis. HB owns IP and receives royalties and licencing fees from Medac for an MSC product for aGvHD which he co-invented. The remaining authors declare that the research was conducted in the absence of any commercial or financial relationships that could be construed as a potential conflict of interest.

## Publisher's Note

All claims expressed in this article are solely those of the authors and do not necessarily represent those of their affiliated organizations, or those of the publisher, the editors and the reviewers. Any product that may be evaluated in this article, or claim that may be made by its manufacturer, is not guaranteed or endorsed by the publisher.
